# Barriers and facilitators to primary care for people living with HIV and diabetes in Harare

**DOI:** 10.4102/phcfm.v16i1.4603

**Published:** 2024-10-08

**Authors:** Rumbidzai Chireshe, Keshena Naidoo, Tawanda Manyangadze

**Affiliations:** 1 Department of Public Health Medicine, Faculty of Nursing and Public Health, University of KwaZulu-Natal, Durban, South Africa; 2 Department of Family Medicine, College of Nursing and Public Health, University of KwaZulu-Natal, Durban, South Africa; 3 Department of Geosciences, Faculty of Sciences and Engineering, Bindura University, Bindura, Zimbabwe

**Keywords:** primary healthcare, HIV, T2DM, availability, readiness, accessibility, SARA, Zimbabwe

## Abstract

**Background:**

People living with human immunodeficiency virus (HIV) and comorbid diabetes mellitus (DM) face significant challenges owing to the complex interplay between these chronic conditions and the need for comprehensive and integrated care. Service availability and readiness for primary care are essential for the health of individuals and populations.

**Aim:**

This study aimed to explore barriers and facilitators to the provision of care to the patients with HIV and T2DM comorbidity.

**Setting:**

The study was conducted at Primary health centres in Harare, Zimbabwe.

**Methods:**

A mixed-methods design was applied.

**Results:**

An audit of primary care facilities identified that there was adequate infrastructure and equipment for HIV and T2DM diagnosis and treatment. However, there are gaps in the availability of essential medicines and supplies, such as test strips for blood glucose monitoring. The assessment also showed that the centres had a chronic shortage of healthcare providers, including doctors, nurses and counsellors, and there was a need for additional training and support for healthcare providers in the management of HIV and T2DM.

**Conclusion:**

The study concludes that the delivery of health services to patients with HIV and T2DM at primary care centres in Harare, Zimbabwe, faces significant challenges. Suggestions included improved resource allocation and multisectoral collaboration to improve the delivery of healthcare services.

**Contribution:**

The research contributes insight into disparities between urban and rural primary care facilities in providing services, emphasizing the need for targeted interventions to bridge gaps and enhance care quality.

## Introduction

In recent years, the global community has come a long way to accomplish the Millennium Development Goals. Success has been achieved through well-thought-out development plans and integrated investments.^1^ This has strengthened health programmes, expanded health coverage and produced targeted improvements in health outcomes in the health sector. Even though we observe these advancements, new data accurately show that progress has been uneven and that there is still room for improvement.^1^ This underscores the urgent need to strengthen health systems and to emphasise the critical importance of primary healthcare and universal health coverage (UHC).

The delivery of health services to patients with human immunodeficiency virus (HIV) infection and type 2 diabetes mellitus (T2DM) at the primary healthcare level is crucial for improving health outcomes. Zimbabwe is one of the countries most affected by the HIV epidemic in sub-Saharan Africa.^2^ According to the Zimbabwe Population-based HIV Impact Assessment (ZIMPHIA 2020), there are an estimated 1.23 million adults living with HIV in Zimbabwe, with an adult HIV prevalence of 12.9%.^3^ The survey also found that HIV prevalence was higher among women than among men (15.3% vs. 10.2%), and that the annual HIV incidence among adults was 0.38%, corresponding to approximately 31 000 new HIV infections per year.^3,4,5^ Type 2 diabetes mellitus is also a growing public health concern in Zimbabwe, as it is estimated that 6.4% of adults have diabetes, which was the fifth leading cause of death in the country in 2019.^6^ A key principle in the management of people with chronic illnesses is continuity of care without treatment disruptions.

Moreover, HIV and T2DM comorbidities pose significant challenges for the health system as they require long-term care, regular monitoring and adherence to medication.^7,8^

One strategy adopted by Zimbabwe to improve the delivery of primary healthcare services, especially for people living with HIV and T2DM, is the establishment of satellite clinics.^9,10^ Satellite clinics are smaller council health facilities linked to a larger polyclinic or hospital that provide basic health services such as HIV testing and counselling, antiretroviral therapy (ART), chronic disease management, maternal and child health and family planning.^9,11^

These satellite clinics are intended to increase the coverage and accessibility of health services, especially for people living in remote, underserved or densely populated areas, by reducing the distance and cost of travel and decongesting the main health facilities.^9,12^ Satellite clinics are also expected to improve the quality and continuity of care by ensuring that people living with HIV and T2DM receive regular follow-up, monitoring and support from trained health workers.^9^

Despite the potential benefits of satellite clinics and polyclinics, there is a lack of evidence regarding how they are perceived and utilised by people living with HIV and T2DM in Zimbabwe. Studies have mainly focused on the health system factors that affect the performance and sustainability of primary care clinics, such as human resources, infrastructure, supplies and supervision. However, there are gaps in service delivery to people who are supposed to benefit from these services and how these gaps influence patients’ health-seeking behaviours and outcomes. Understanding the availability and readiness of primary health centres (PHCs) for primary care service delivery is important for identifying the strengths and weaknesses of the current model and informing the design and implementation of effective and responsive interventions.

The World Health Organization (WHO) Global Action Plan for the Control of non-communicable diseases (NCDs) 2013–2020 states that countries should adopt person-centred PHC and UHC to enhance their health systems and fight NCDs.^13^ Most Africans receive their medical care at government-funded hospitals or on an out-of-pocket basis, despite efforts by some African nations, including Ghana and Nigeria, to achieve UHC through universal health insurance systems.^14,15^ Multiple visits and services from the health sector further compromise individuals with multiple morbidities.

According to a study conducted in Zimbabwe, people with HIV and T2DM had difficulty accessing healthcare because of a variety of issues, such as lengthy wait times, expensive bills and lack of understanding about the management of their conditions.^16,17^

Another study conducted in sub-Saharan Africa found that healthcare providers faced challenges in the delivery of health services, including limited resources, high patient loads and inadequate support from the healthcare system.^18^

To ensure that these services meet the demands of the patients, it is necessary to evaluate their readiness and availability. This study aimed to assess the availability and readiness of health services for patients with HIV and T2DM at eight primary care centres in Harare using the WHO Service Availability and Readiness Assessment (SARA) methodology and structured interviews. The WHO SARA methodology has been widely used to assess the availability and readiness of health services in various settings.^19^

## Research methods and design

### Study design

This study employed a mixed-methods approach to explore the preparedness of primary healthcare facilities to deliver care to patients with HIV and T2DM comorbidities. An audit of services at primary care clinics was conducted using a validated data collection tool (WHO) (SARA tool).^20^ The strengths and weaknesses of the healthcare services were further explored through semi-structured interviews with key informants.

The mixed-methods approach was chosen to provide a multifaceted perspective on the study topic and explore potential strategies to strengthen current health systems.

### Study setting

This study explored the primary care services provided to patients with HIV and T2DM in Harare, the capital city of Zimbabwe. Clinics funded by the government and non-governmental organisations (NGOs) provide primary healthcare services to approximately 2.5m people in urban and rural communities in Harare.^21^

The range of services provided at PHCs includes HIV screening, diabetes and hypertension management, management of acute and chronic conditions and health promotion. The facilities are mostly staffed by professional nurses and managed by nurse managers (sisters-in-charge or matrons).

### Sampling

Eight of the 12 primary healthcare facilities in Harare were purposely selected, of which six were located in urban communities and two in rural communities.

### Data collection

Data were collected between January 2022 and December 2023 by a researcher and trained research assistants. The SARA assessment was conducted through observations, checklists and a review of policy documents and patient records.^22^ The checklist collected information on the demographic data and training of staff, as well as the presence or absence of guidelines, basic equipment relevant to diabetes and HIV care, diagnostic services, essential medicines, community services, education and counselling.

Key informants at each study site (facility managers and healthcare providers) were identified and invited to participate. Participants were informed of the study by the researcher, and written informed consent was obtained. Participants were recruited until data saturation was reached.

The researcher conducted the interviews in English, aided by an interview guide, to elicit participants’ perceptions of the quality of primary healthcare services for patients with HIV and T2DM co-morbidity and to identify possible barriers and facilitators of integrated care. The interviews were audio-recorded and transcribed before analysis.

### Data analysis

Observational data and data extracted from facility records were captured using a data collection tool. Data were collated and analysed using the Statistical Package for Social Science (SPSS). Data relevant to the study objectives were reported using simple descriptive statistics such as frequencies and percentages.

All of the transcripts of the interviews were read thoroughly to get a sense of the whole, and content analyses were conducted for each transcript, all of which were done by the researcher. Similar concepts were clustered together, data were integrated and synthesised into a descriptive structure, and codes were created. The themes derived from these codes were grouped into domains.

### Data collection instrument

The WHO SARA is a validated tool used to assess the availability and readiness of health services in six areas: infrastructure, equipment, medicines, supplies, healthcare providers and patient information (Online Appendix 1). Each domain contains a specific number of tracer items.^23^ This tool was chosen because it has been validated globally, and it offers a comprehensive framework for assessing the availability and readiness of health services for specific conditions. There is a multi-domain assessment that evaluates various aspects of service delivery such as staffing, infrastructure and medicines. The SARA provides a reliable and well-established framework for evaluating service delivery generally and for specific populations, such as patients with HIV and T2DM comorbidities.

### Ethical considerations

The researchers obtained ethics approval from the University of KwaZulu-Natal Biomedical Research Ethics Committee (Approval number: BREC/00003160/21) and the Medical Research Council of Zimbabwe (Approval number: MRCZ/A/2821) prior to data collection. Written informed consent was obtained from the participants of the semi-structured interviews, and their confidentiality and anonymity were maintained throughout the study.

## Results

### Staffing and infrastructure

The availability and readiness of health services for patients with HIV and T2DM were reported according to six domains: staffing levels, training, availability of guidelines, infrastructure, equipment and essential medicines.

#### Staffing levels

**FIGURE 1:**
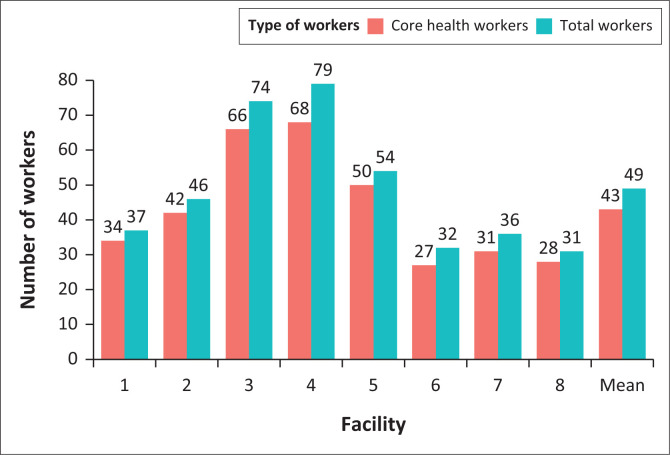
Staff employed at primary care facilities.

Figure 1 displays the staffing levels at the six urban (numbers 1–6) and two rural clinics (number 7 and 8). Generally, urban primary care facilities have larger staff sizes compared to rural primary care facilities.

#### Staff training, equipment and essential medication

The findings on the training of staff, availability of guidelines, infrastructure, equipment and essential medicines are reported in Table 1. Training was defined as health providers with training on HIV Vertical Transmission Programme, voluntary counselling and testing, HIV/AIDS prevention, care and management for adolescents, ART prescription and management, clinical management of HIV/AIDS, diagnosis and management of diabetes; diagnosis and management of cardiovascular diseases such as hypertension, diagnosis and management of chronic respiratory diseases. The presence of standard precautions was audited and included standard precautions for infection prevention and pre-and post-exposure HIV counselling and testing. Basic equipment requirements included adult scale, thermometer; stethoscope, sphygmomanometer and BP cuff, light source, intravenous infusion kits, oxygen concentrators, oxygen cylinders and availability of oxygen. Facilities were expected to provide on-site testing of haemoglobin, blood glucose, HIV rapid tests, general microscopy and urine dipstick. Essential medication required for chronic diseases included metformin, glibenclamide, amitriptyline, atenolol, captopril, co-trimoxazole paracetamol and ART.

**TABLE 1:** Training of staff, availability of guidelines, infrastructure, equipment and essential medicines.

Domain	Percentage of standards met											
	Facility (Urban)								Facility (Rural)			
	1	2	3	4	5	6	Mean	s.d.	1	2	Mean	s.d.
Training	75.0	62.0	87.0	87.0	87.0	65.0	79.0	10.0	75.0	75.0	75.0	0.00
Standard precautions	86.0	86.0	100.0	100.0	100.0	71.0	94.0	7.0	71.4	86.0	81.0	9.0
Basic equipment	78.0	89.0	89.0	67.0	78.0	67.0	78.0	10.0	66.7	56.0	61.0	8.0
Laboratory	60.0	60.0	60.0	60.0	60.0	40.0	57.0	13.0	40.0	40.0	40.0	0.0
Medicines	86.0	71.0	86.0	71.0	57.0	57.0	71.0	13.0	57.1	71.0	64.0	10.0

s.d., standard deviation.

Across almost all domains, urban facilities scored higher than rural facilities. Training of staff in urban facilities was measured to be a mean of 79.2% (range 62.5–87.5) while the mean in the rural facilities was 75.0%. The mean for the presence of standard precautions was 93.8% (range 71.4–100) in urban facilities and 81.3% (range 71.4–85.7). The availability of basic equipment ranged from 55.6% to 89.9%, with the two rural facilities scoring the lowest. The ability of rural facilities to perform basic on-site testing was extremely poor at 40%. Urban facilities were only marginally better at a mean of 56.7% (s.d. 12.8).

#### Basic amenities

The availability of basic amenities required for clinics to provide essential services includes communication, ambulance, power supply, water and sanitation, privacy to examine patients and computer with internet access. Clinic access to each of the required amenities is shown in Table 2.

**TABLE 2:** Availability of basic amenities.

Amenity	Percentage (%) score of availability of amenities											
	Facility (Urban)								Facility (Rural)			
	1	2	3	4	5	6	Mean	s.d.	1	2	Mean	s.d.
Communication	67.0	67.0	67.0	67.0	67.0	67.0	67.0	0.0	33.0	67.0	50.0	24.0
Emergency transport	33.0	33.0	100.0	33.0	33.0	33.0	44.0	27.0	33.0	67.0	50.0	24.0
Power supply	86.7	71.0	71.0	57.0	57.0	71.0	69.0	11.0	71.0	57.0	64.0	10.0
Water sources	100.0	100.0	100.0	100.0	100.0	100.0	100.0	0.0	100.0	100.0	100.0	0.00
Sanitation services	80.0	60.0	80.0	80.0	80.0	60.0	73.0	10.0	40.0	60.0	50.0	10.0
Privacy	100.0	0.0	100.0	0.0	0.0	100.0	50.0	55.0	100.0	0.0	50.0	71.0
Computer	100.0	100.0	100.0	100.0	100.0	50.0	92.0	20.0	50.0	50.0	50.0	0.00
**Mean at facility**	**82.0**	**62.0**	**88.0**	**62.0**	**62.0**	**69.0**	**71.0**	**-**	**61.0**	**57.0**	**-**	**-**

s.d., standard deviation.

All, except one rural facility, had two of the three communication options (i.e. functioning landline; functioning cellular telephone; functioning short-wave radio for radio calls). One rural facility only had one means of communication, and none of the clinics had all three.

Facilities were evaluated for their preparedness to transport patients with emergencies to hospital according to the following three criteria: functional ambulance/vehicle for emergency transportation; access to an ambulance/vehicle for emergency transport and fuel for the ambulance/emergency vehicle. Only one of the sites had all three, six had two out of three and one of the rural sites had only one.

The audit of clinic power supply included whether facilities had access to electricity from any source (e.g. electricity grid, generator, solar, etc.), central supply of electricity as a main source, secondary or backup source of electricity, electricity available at all times from the main or a backup source in previous week, functional generator, fuel/charged battery or functional solar system. Urban facilities scored 69.1% on average (range 57.1–85.7) while rural sites 57.1% and 71.4% for availability of reliable power supply.

Access to safe water consisted of an inspection of the availability of safe water for the facility and a supply of safe water available within the premise. All facilities were fully compliant (100%) with the requirements regarding safe water supply and access.

The audit of sanitation consisted of an inspection regarding waste management practices for sharps waste (such as needles or blades), medical waste other than sharps (such as used bandages), functional incinerator, fuel for the incinerator and toilet (latrine) on premises in functioning condition. Urban clinics scored a mean of 73.3% (s.d. ±10.3) for sanitation requirements, while rural facilities scored a much lower mean of 50% (s.d. ±10).

Only half of the facilities had consulting rooms that ensured privacy for patient consultations. Five of the eight sites had a functioning computer and access to email or internet, and three only had one of the two.

#### Specific services for patients with HIV and chronic non-communicable disease

The readiness of facilities to deliver specific services is shown in Table 3.

**TABLE 3:** Targeted services for people living with HIV and type 2 diabetes mellitus.

Area of care	Number (*n*) and percentage (%) of items or standards met at the facility (Responses = yes)											
	Facility (Urban)								Facility (Rural)			
	1	2	3	4	5	6	Mean	s.d.	1	2	Mean	s.d.
HIV testing and counselling	100.0	75.0	100.0	100.0	100.0	100.0	96.0	10.0	100.0	75.0	87.0	18.0
HIV AIDS care and support	100.0	86.0	71.0	100.0	71.0	86.0	86.0	13.0	86.0	71.0	79.0	10.0
Antiretroviral therapy	83.0	100.0	100.0	100.0	83.0	83.0	92.0	9.0	100.0	100.0	100.0	0.00
PMTCT	100.0	100.0	91.0	91.0	91.0	100.0	97.0	5.0	100.0	82.0	91.0	13.0
T2DM#	83.0	83.0	100.0	100.0	91.0	83.0	92.0	9.0	100.0	83.0.	92.0	12.0

T2DM, type 2 diabetes mellitus; PMTCT, Prevention-of-mother-to-child-transmission; s.d., standard deviation.

# , non-communicable diseases.

Almost all facilities (range 75% – 100%) possessed the minimum required resources for HIV screening, prevention and treatment, which were the guidelines on HIV testing, guidelines on HIV/AIDS counselling, HIV rapid diagnostic test and male condoms. Facilities also demonstrated strong capacity (83.3% – 100%) to treat patients with HIV and opportunistic infections, evidenced by the availability of guidelines for HIV/AIDS opportunistic infection treatment and care, TB testing, intravenous infusion kit, co-trimoxazole and first-line TB medications. Scores ranged from 83.38% to 100%, indicating that facilities had strong readiness to manage patients on antiretroviral treatment. The indicators for readiness included the availability of first-line antiretrovirals Tenofovir Disoproxil Fumarate (TDF) + emtricitabine (FTC) + Efavirenz (EFZ)/Nevirapine (NVP) and capacity to perform investigations such as CD4, viral load, blood urea levels and liver function tests.

Facilities also scored highly in the preparedness to provide prevention of mother-to-child transmission of HIV. Between 81.1% and 100% of facilities provided HIV counselling and testing services to HIV-positive pregnant women and infants, prophylaxis to HIV-exposed infants, infant feeding counselling, nutritional counselling for HIV-positive pregnant women and their infants, family planning, training in prevention of mother-to-child transmission (PMTCT) programme and training in infant and young child feeding.

Facilities also possessed satisfactory readiness to care for patients with chronic non-communicable diseases, with scores from 81.3% to 100%. Care of patients with T2DM included guidelines and resources for diagnosing and managing T2DM, as well as specific training of staff.

Table 3 shows that both the urban and rural healthcare facilities performed equally well in meeting the NCD standards assessed, with both achieving an average of 91.67% compliance.

### Healthcare providers’ perspectives on the delivery of health services for patients with HIV and type 2 diabetes mellitus

Interviews with healthcare providers revealed that they faced challenges in the delivery of health services that included staff shortages, lack of training, limited resources, high patient loads and inadequate support from the healthcare system.

#### Human resources

Participants highlighted the challenges of staffing shortages and the lack of training affected the ability of participants to provide optimal care. Several participants reported that they only occasionally provided counselling to patients with diabetes because of staff shortages. Others simply give patients posters or leaflets (if available) so they can read if they can do so. Participants identified the main reasons for the lack of staff, supporting materials and time:

‘We are facing healthcare worker shortages at the moment with this massive relocation of healthcare workers to European countries in search for greener pastures, nurse patient ratio at the moment is unexplainable.’ (41 year old, Sister-in-charge, Clinic 3)

Participants expressed dissatisfaction with by the absence of guidelines in the facilities and inadequate training in HIV and diabetes during the previous 2 years. Many participants expressed dissatisfaction with the lack of diabetes-related in-service training opportunities. Many stated that the information they learned during pre-service training and education served as the foundation for their practice. They added that the absence of an official copy of the diabetes management guidelines in each of their facilities exacerbated their lack of knowledge:

‘We are very unfortunate, in some of our facilities there are no guidelines, so we are treating patients with our out-dated knowledge that we acquired back in college.’ (54 year old, Sister-in-charge, Clinic 4)

One participant observed that certain conditions, such as HIV, are the focus of education campaigns and counselling sessions; however, patients with diabetes do not receive the same benefits because their disease progresses slowly and silently. He also discussed the funders’ lack of interest in providing financial support for the initiative:

‘Infectious diseases tend to grab everyone’s attention quickly which will then result in non-communicable diseases being neglected.’ (57 year old, Matron, Clinic 1)

#### Infrastructure

Participants expanded on the challenges with inadequate medicines and laboratory tests:

‘We go for a long time without medical supplies for non-communicable diseases, patients have to go purchase their supply from private pharmacies, take their blood samples for tests like Hb1AC to private laboratories which is expensive.’ (38 year old, Sister-in-charge, Clinic 7)

In addition, participants cited difficulty in accessing healthcare, lengthy waiting times, exorbitant hospital bills and a lack of HIV and T2DM management knowledge:

‘Patients must receive treatment for HIV from the HIV clinic, and from there, they must go to the outpatient department for the treatment of diabetes, which is time consuming and expensive, especially if the review appointment dates are different. They receive free treatment for HIV care and management, and they have to pay for diabetes management in the outpatient department. A lot of these patients with comorbidities miss diabetes appointments.’ (48 year old, Sister-in-charge, Clinic 7)

There were also positive perceptions expressed by participants of the services provided by their facilities, as supported by the following statements:

‘It has never happened that we go out of stock for ARVs in the OI Clinic. There is constant supply of medical supplies, patients get medical services at no cost at all and services like counselling are available.’ (44 year old, Sister-in-charge, Clinic 2)

and;

‘The clinic staff is a well-resourced team equipped with additional finance, time and team members with improved electronic communication for better record keeping.’ (54 year old, Sister-in-charge, Clinic 6)

Some participants identified that integration for care would facilitate service delivery:

‘Integration of care foster commitment and will be easy to monitor care quality and performance across board.’ (40 year old, Sister-in-charge, Clinic 8)

## Discussion

This study evaluated the preparedness of primary care facilities in Harare, Zimbabwe to deliver services to people with HIV and T2DM. The findings indicate that primary care facilities in Harare were inadequately prepared to care for patients with complex comorbid conditions such as HIV and T2DM, scoring below the 90% readiness target.^24^

A notable discordance in staffing levels was noted between rural and urban primary care facilities. Rural facilities had less staff and staff with less training, which poses additional challenges to the provision of care to patients in rural areas with HIV and T2DM. A possible reason for lower staff numbers and levels of training in rural clinics may be the attrition of staff from rural to urban areas. As a result, greater focus is required on recruiting and retaining staff to work in rural areas.

Training readiness in both rural and urban facilities was 75%, suggesting that healthcare providers may have gaps in knowledge and skills and may provide suboptimal care of patients. Patients with comorbid HIV and type 2 diabetes require health providers with awareness of managing patients with medical complexities in order to co-ordinate their clinical care. Targeted training is required to equip health providers to provide integrated care, avoid adverse drug-drug interactions, support patient self-management and address psychosocial barriers to care.

Significant gaps were identified in the infrastructure and the availability of amenities at all facilities required to deliver services. Basic equipment was absent in many sites, particularly in those in rural areas. Another area of serious concern is the inability of many facilities to perform basic investigations on-site. Laboratory readiness was particularly low in rural areas (40%) as well as urban facilities (57%). This low diagnostic capacity is consistent with the findings of other African studies.^25,26,27,28,29^ Inadequate laboratory infrastructure and services in rural areas could lead to challenges in the timely diagnosis and monitoring of HIV and diabetes, adjustment of medications and treatments based on laboratory test results and detection and management of complications associated with the two conditions. According to the WHO, essential laboratory services should be available and accessible to at least 80% of the population.^24^ For the care of patients with complex conditions, such as HIV and diabetes, an even higher level of laboratory readiness may be desirable to ensure comprehensive monitoring and management. Sub-optimal levels of equipment and investigations also heighten the importance of staff training to advocate for quality patient care.

Primary care facilities demonstrated strong systems to counsel and screen for HIV and T2DM. However, the non-availability of essential medications is extremely alarming. Treatment disruption among patients on antiretroviral treatment can result in decreased levels of viral suppression and potential drug resistance. Zimbabwe, like many other African countries, has not managed to attain the 95-95-95 targets. Increased efforts are needed to ensure a consistent supply of essential medicines in all primary care facilities to improve adherence to treatment. Unsurprisingly, rural sites fared worse than their urban counterparts regarding the availability of essential medication. Only 64% of the 14 essential medicines were available in rural clinics, whereas 71% were available in urban clinics. Of note, there was scarcity of essential medications such as insulin in primary care. The lack of these essential medicines can lead to life-threatening complications.^25,30^ The study findings reflect the situation in other African countries. A similar audit across Mozambique found the availability of diabetes medicines to be 58% in rural facilities versus 77% in urban facilities.^20,31^

All facilities had access to safe water but lacked other amenities such as private consultation rooms and emergency patient transport. Facilities in rural sites had the least resources with suboptimal sanitation, limited access to computers, internet access and reliable communication means. The absence of emergency patient transport could account for avoidable deaths from treatable conditions.

The findings are concordant with reports from health users of the difficulties in accessing care. Similar issues have been documented in other low- to middle-income countries, such as Zambia, Tanzania, Kenya, Malawi, Namibia, Nepal, Rwanda, Bangladesh and Haiti.^30,32,33,34^

Facility managers and providers interviewed suggested the following solutions in addressing these issues. Supply chain and logistics management should be strengthened by investing in upgrading supply chain infrastructure, including transportation, storage and inventory management systems; implementing digital technologies (e.g. electronic logistics management information systems) to enhance supply chain visibility and responsiveness and establishing robust forecasting and procurement processes to better anticipate and meet the demands of remote health facilities.

Enhancing quality improvement initiatives was another solution offered by key informants. This could be achieved by developing and implementing standardised clinical protocols and care pathways for integrated HIV and diabetes management, providing continuous training and mentorship programmes to build the capacity of healthcare workers in remote areas and implementing regular supportive supervision and performance monitoring to identify and address gaps in quality of care. Another solution was to adopt decentralised and community-based service delivery models by expanding the role of community health workers and peer supporters to extend the reach of services in remote areas, establishing mobile health clinics or outreach teams to periodically visit hard-to-reach communities and empowering patients and communities to actively participate in the planning and management of local health services.

Leveraging digital health technologies via utilisation of telemedicine and eHealth solutions to provide remote consultation, diagnosis and treatment support; deploying health tools for real-time monitoring of stock levels, patient adherence and service utilisation and integrating digital data systems to improve health information management and decision-making at all levels. Another solution is to foster multi-stakeholder collaboration by engaging with local governments, community organisations and private sector partners to mobilise additional resources and expertise, promote cross-sectoral coordination to address the social determinants of health in remote communities and strengthen community-facility linkages to enhance patient engagement and support.

Building the capacity of healthcare providers is crucial for improving the health systems in these regions. Approaches in low- and middle-income countries (LMICs) often emphasise task shifting and training of healthcare personnel.^35^ In this study, the mean number of core health staff was 43%, which is less than half, leading to increased workload for health workers. Urban clinics have better staffing than rural clinics.

In diabetes management, task shifting and training have yielded positive results such as improved diagnosis and management adherence, early screening, reduced uncontrolled diabetes cases, fewer hospitalisations because of acute metabolic complications, consistent drops in glycosylated haemoglobin levels and better identification and referral of inadequately controlled cases.^36^ Despite these successes, many patients with diabetes do not receive the recommended care, which healthcare providers attribute to financial problems or different appointment schedules. Group counselling sessions were occasionally available to educate the patients on medication adherence, lifestyle changes and complications. Providers cited several barriers, including staff shortages, time constraints, long distances, silent disease progression and lack of supportive supplies.

Guidelines are vital in primary health care, and their absence can severely impair facility functionality. To be effective, guidelines must be user-friendly, widely disseminated and implemented at all levels of the healthcare system, including primary healthcare. Given the generally poor educational background of practitioners in peripheral facilities, it is imperative to distribute copies of the guidelines throughout these remote health facilities to positively impact patient management.^37,38^ Guidelines help to maintain care quality, harmonise practice with current research and reduce provider frustration when managing complex cases.

Financial barriers also hinder diabetes service readiness despite Zimbabwe’s policy of providing free public sector services at the point of care.^39^ Frequent stockouts, poor quality of care and slow programme rollouts in remote areas because of funding shortages remain significant obstacles.

## Conclusion

The study concludes that the delivery of health services to patients with HIV and T2DM at primary care clinics in Harare, Zimbabwe, faces significant challenges. There is a need for increased attention to rural health facilities to address the health disparities faced by rural communities. Proactive recruitment and retention of staff as well as active training of health providers would help improve the quality of care of patients. Further investigation is required into the lack of essential medicines and strategies to ensure treatment for people living with chronic diseases such as HIV and T2DM.

This study also underscores the importance of addressing the challenges faced by healthcare providers to ensure that they can provide quality care to patients. The following are the recommendations from this study: enhancing the distribution of resources to primary care facilities to close the gaps in the supply of necessary medications and supplies. Patient education to increase patients’ understanding of HIV and T2DM management and to encourage treatment adherence, as well as training and support for healthcare professionals in managing HIV and T2DM, can improve the quality of care provided to patients.

## References

[1] World Health Organization. WHO global strategy on people-centered and integrated healths: Interim report. Geneva: World Health Organization; 2015.

[2] Abas M, Nyamayaro P, Bere T, et al. Feasibility and acceptability of a task-shifted intervention to enhance adherence to HIV medication and improve depression in people living with HIV in Zimbabwe, a low income country in sub-Saharan Africa. AIDS Behav. 2018;22:86–101. 10.1007/s10461-016-1659-4 28063075 PMC5758696

[3] ZIMPHIA. Zimbabwe population based HIV impact assessment. Harare: Ministry of health and child care; 2020.

[4] Memiah P, Nkinda L, Majigo M, et al. Prevalence of glucose metabolic disorders and associated inflammatory markers among people living with HIV in sub-Saharan Africa. Metabol Syndr Relat Disord. 2022;20(1):20–28. 10.1089/met.2021.0054 35179982

[5] Nkinda L, Patel K, Njuguna B, et al. C-reactive protein and interleukin-6 levels among human immunodeficiency virus-infected patients with dysglycemia in Tanzania. BMC Endocr Disord. 2019;19(1):1–8. 10.1186/s12902-019-0407-y 31331321 PMC6647154

[6] Arokiasamy P, Salvi S, Selvamani Y. Global burden of diabetes mellitus: Prevalence, pattern, and trends. In: Haring R, Kickbusch I, Ganten D, Moeti M, editors. Handbook of global health. London: Springer Nature, 2021; p. 495–538.

[7] Godongwana M, Wet-Billings D, Milovanovic M. The comorbidity of HIV, hypertension and diabetes: A qualitative study exploring the challenges faced by healthcare providers and patients in selected urban and rural health facilities where the ICDM model is implemented in South Africa. BMC Health Serv Res. 2021;21(1):1–15. 10.1186/s12913-021-06670-3 34217285 PMC8254615

[8] Chireshe R, Manyangadze T, Naidoo K. Diabetes mellitus and associated factors among HIV-positive patients at primary health care facilities in Harare, Zimbabwe: A descriptive cross-sectional study. BMC Prim Care. 2024;25(1):28. 10.1186/s12875-024-02261-3 38221613 PMC10789024

[9] Ray S, Goronga T, Chigiya PT, Madzimbamuto FD. Climate change, disaster management and primary health care in Zimbabwe. Afr J Prim Health Care Fam Med. 2022;14(1):1–3. 10.4102/phcfm.v14i1.3684 PMC9575364 36226938

[10] Nkomo T. Optimising primary health care facility location: A case of Gweru District. Gweru: Midlands state university; 2018.

[11] Link A, Tshimanga M, Cochrane B, Kasprzyk D. High satisfaction among patients at HIV clinics in Harare, Zimbabwe: A time and motion evaluation and patient satisfaction study. Int J Qual Health Care. 2023;35(2):mzad030. 10.1093/intqhc/mzad030 37294882 PMC10256183

[12] World Health Organization. Adolescent friendly health services for adolescents living with HIV: From theory to practice, December 2019: Technical brief. Geneva: World Health Organization; 2019.

[13] Chestnov O. World Health Organization global action plan for the prevention and control of noncommunicable diseases. Geneva: World Health Organization; 2013.

[14] Umeh CA. Challenges toward achieving universal health coverage in Ghana, Kenya, Nigeria, and Tanzania. Int J Health Plan Manage. 2018;33(4):794–805. 10.1002/hpm.2610 30074646

[15] Amu H, Dickson KS, Kumi-Kyereme A, Darteh EKM. Understanding variations in health insurance coverage in Ghana, Kenya, Nigeria, and Tanzania: Evidence from demographic and health surveys. PLoS One. 2018;13(8):e0201833. 10.1371/journal.pone.0201833 30080875 PMC6078306

[16] Singh S, Kirk O, Jaffar S, et al. Patient perspectives on integrated healthcare for HIV, hypertension and type 2 diabetes: A scoping review. BMJ Open. 2021;11(11):e054629. 10.1136/bmjopen-2021-054629 PMC8596045 34785559

[17] Roets L, Mangundu M, Janse van Rensberg E. Accessibility of healthcare in rural Zimbabwe: The perspective of nurses and healthcare users. Afr J Prim Health Care Fam Med. 2020;12(1):1–7. 10.4102/phcfm.v12i1.2245 PMC7284155 32501024

[18] Sande S, Zimba M, Mberikunashe J, Tangwena A, Chimusoro A. Progress towards malaria elimination in Zimbabwe with special reference to the period 2003–2015. Malar J. 2017;16:1–13. 10.1186/s12936-017-1939-0 28738840 PMC5525350

[19] World Heath Organization. Service availability and readiness assessment (SARA) reference manual. Geneva: World Health Organization; 2015.

[20] Iyengar S, Van den Ham R, Suleman F. Medicine prices in Africa. Medicine price surveys, analyses and comparisons. London: Elsevier, Academic Press; 2019, p. 85–111.

[21] Zimbabwe National Statistics Agency. 2022 population & housing census – Preliminary 2022 [homepage on the Internte]. [cited 2024 Jan 15]. Available from: https://zimbabwe.opendataforafrica.org/anjlptc/2022-population-housing-census-preliminary.

[22] Scholz S, Ngoli B, Flessa S. Rapid assessment of infrastructure of primary health care facilities–A relevant instrument for health care systems management. BMC Health Serv Res. 2015;15:1–10. 10.1186/s12913-015-0838-8 25928252 PMC4421986

[23] World Health Organization. Service availability and readiness assessment (SARA): An annual monitoring system for service delivery: Reference manual. Geneva: World Health Organization; 2013.

[24] World Health Organization. Action framework to advance universal access to safe, effective and quality-assured blood products 2020–2023. Geneva: World Health Organization; 2020.

[25] Amberbir A, Lin SH, Berman J, et al. Systematic review of hypertension and diabetes burden, risk factors, and interventions for prevention and control in Malawi: The NCD BRITE Consortium. Glob Heart. 2019;14(2):109–118. 10.1016/j.gheart.2019.05.001 31324364

[26] Gauld R, Blank R, Burgers J, et al. The World Health Report 2008–primary healthcare: How wide is the gap between its agenda and implementation in 12 high-income health systems? Healthc Policy. 2012;7(3):38. 10.12927/hcpol.2013.22778 23372580 PMC3298021

[27] Assayed A, Muula A, Nyirenda M. The quality of care of diabetic patients in rural Malawi: A case of Mangochi district. Malawi Med J. 2014;26(4):109–114. 26167259 PMC4325344

[28] Chikowe I, Mwapasa V, Kengne AP. Analysis of rural health centres preparedness for the management of diabetic patients in Malawi. BMC Res Notes. 2018;11(1):1–6. 10.1186/s13104-018-3369-7 29720279 PMC5932777

[29] Pfaff C, Malamula G, Kamowatimwa G, et al. Decentralising diabetes care from hospitals to primary health care centres in Malawi. Malawi Med J. 2021;33(3):159–168. 10.4314/mmj.v33i3.3 35233273 PMC8843181

[30] Jigjidsuren A, Byambaa T, Altangerel E, et al. Free and universal access to primary healthcare in Mongolia: The service availability and readiness assessment. BMC Health Serv Res. 2019;19:1–12. 10.1186/s12913-019-3932-5 30786897 PMC6381625

[31] Beran D, Ewen M, Lipska K, Hirsch IB, Yudkin JS. Availability and affordability of essential medicines: Implications for global diabetes treatment. Curr Diabetes Rep. 2018;18:1–10. 10.1007/s11892-018-1019-z 29907884

[32] Mutale W, Bosomprah S, Shankalala P, et al. Assessing capacity and readiness to manage NCDs in primary care setting: Gaps and opportunities based on adapted WHO PEN tool in Zambia. PLoS One. 2018;13(8):e0200994. 10.1371/journal.pone.0200994 30138318 PMC6107121

[33] Leslie HH, Spiegelman D, Zhou X, Kruk ME. Service readiness of health facilities in Bangladesh, Haiti, Kenya, Malawi, Namibia, Nepal, Rwanda, Senegal, Uganda and the United Republic of Tanzania. Bull World Health Organ. 2017;95(11):738. 10.2471/BLT.17.191916 29147054 PMC5677617

[34] Bintabara D, Ngajilo D. Readiness of health facilities for the outpatient management of non-communicable diseases in a low-resource setting: An example from a facility-based cross-sectional survey in Tanzania. BMJ Open. 2020;10(11):e040908. 10.1136/bmjopen-2020-040908 PMC7661355 33177143

[35] Cundale K, Wroe E, Matanje-Mwagomba BL, et al. Reframing noncommunicable diseases and injuries for the poorest Malawians: The Malawi National NCDI Poverty Commission. Malawi Med J. 2017;29(2):194–197. 10.4314/mmj.v29i2.22 28955432 PMC5610295

[36] Distiller L, Brown M, Joffe B, Kramer B. Striving for the impossible dream: A community-based multi-practice collaborative model of diabetes management. Diabet Med. 2010;27(2):197–202. 10.1111/j.1464-5491.2009.02907.x 20546264

[37] Mayega RW, Guwatudde D, Makumbi FE, et al. Comparison of fasting plasma glucose and haemoglobin A1c point-of-care tests in screening for diabetes and abnormal glucose regulation in a rural low income setting. Diabetes Res Clin Pract. 2014;104(1):112–120. 10.1016/j.diabres.2013.12.030 24456993

[38] Katende D, Mutungi G, Baisley K, et al. Readiness of Ugandan health services for the management of outpatients with chronic diseases. Trop Med Int Health. 2015;20(10):1385–1395. 10.1111/tmi.12560 26095069 PMC4758403

[39] Biswas T, Haider MM, Gupta RD, Uddin J. Assessing the readiness of health facilities for diabetes and cardiovascular services in Bangladesh: A cross-sectional survey. BMJ Open. 2018;8(10):e022817. 10.1136/bmjopen-2018-022817 PMC6252707 30385441

